# Competence profiles in undergraduate dental education: a comparison between theory and reality

**DOI:** 10.1186/s12903-017-0403-4

**Published:** 2017-07-11

**Authors:** Sebastiaan Koole, Shani Van Den Brulle, Véronique Christiaens, Wolfgang Jacquet, Jan Cosyn, Hugo De Bruyn

**Affiliations:** 1 Department of Periodontology and Oral Implantology, Dental School, Ghent University, Ghent, Belgium; 2 Department of Periodontology and Oral Implantology, Vrije Universiteit Brussel, Brussels, Belgium; 3 Department of Prosthodontics, School of Dentistry, Malmö University, Malmö, Sweden

**Keywords:** Dental education (mesh), Clinical competence (mesh), Competency-based education (mesh), Undergraduate dental education, Curriculum development, Questionnaire, Clinical reality

## Abstract

**Background:**

Competence profiles are purposed to provide a blueprint in support to develop and/or benchmark the learning outcomes of undergraduate dental curricula. This study aims to investigate whether a competence profile as proposed by academic- and clinical experts is able to represent the real clinical reality.

**Methods:**

A questionnaire was developed including questions about gender and age, perception about required competences, and educational organisation and was distributed among Flemish dentists via email and on paper during a symposium. The data was analysed using descriptive statistics, Chi-square and non-parametric Mann-Whitney U-tests.

**Results:**

A total of 312 questionnaires were completed (=6.5% of dentist population, with similar gender and age characteristics). All competences in the European competence profile were rated between 7.2 and 9.4 on a 10-point scale. In dentists under 50 years, females rated the importance of identifying/managing anxiety and abnormal patient behaviour; and promoting/improving oral health as significantly higher than males. In dentists of 50 years and above, females rated 8 competences significantly higher than males, including obtaining/recording a complete history; identifying/managing anxiety and abnormal patient behaviour; obtaining/interpreting radiography; identifying temporomandibular and associated disorders; identifying orthodontic needs; awareness of own limitations/when to refer; managing dental urgencies; and basic-life-support/defibrillation. Clinical practice management was most frequently reported as additional competence to address in dental education. Furthermore, the respondents suggested an undergraduate dental curriculum based on 34% theoretical education, 26% preclinical skills training, and 40% clinical education and 86% agreed with a duration of 5 years. Finally, the respondents also illustrated the dynamic nature of dentistry including a reduction of amalgam fillings, a shift from individual practice to group practices, an increased administrative load, and more assertive patients.

**Conclusion:**

Findings in the present study suggest the validation of the proposed competences for graduating European dentists within the clinical reality of dental professionals in daily practice. Nevertheless, the results have also demonstrated heterogeneity regarding gender and age within the dentist population and emphasised a continuously evolving dental profession and required competences. Hence, to maintain high quality of dental care, a strategy should be developed in which dental curricula are continuously benchmarked against an evolving clinical reality.

**Electronic supplementary material:**

The online version of this article (doi:10.1186/s12903-017-0403-4) contains supplementary material, which is available to authorized users.

## Background

Current daily dental practice is characterised by a continuous evolvement of knowledge and treatment options [1, 2], the presence of an increasing amount of assertive patients with high expectations [3] and a growing need for multidisciplinary collaboration [4–6]. To ensure high quality of care, educational institutes should develop dentists equipped with the competences to successfully cope with the challenges in every day practice.

To transform students into dentists, multiple teaching strategies are used by educational institutes ranging from theoretical education to pre-clinical education and clinical education [7]. Within this transformation process, assessment has a crucial role to evaluate the efficiency of provided education and whether students have achieved the intended learning outcomes. In order to develop effective dental education both teaching- and assessment strategies should be aligned with learning outcomes based on the requirements derived from clinical reality [8].

In support of this process, all over the world multiple competence profiles for graduated dentists have been proposed as instruments for curriculum development [9–13]. Despite the societal differences between regions, these profiles share common ground in terms of core competences leading to safe and independent practice of dentistry [14]. These core competences include professionalism; communication and social skills; patientcare including assessment, diagnosis and treatment planning; prevention and health promotion; and scientific and clinical knowledge handling.

Competence profiles are purposed to provide a blueprint in support to develop and/or benchmark the learning outcomes of undergraduate dental curricula. Often they are authored by a group of leading academic- and clinical experts and represent the agreement between their different perspectives on education and dental practice. As a result of continuous societal change, this clinical situation is in constant alteration. Hence, experts have acknowledged the need for frequent updates of competence profiles [11].

The validity of a competence profile is grounded in its ability to represent the real clinical situation. The present study aims to investigate whether the competences described in a profile for newly graduated dentists, as proposed by academic- and clinical experts, are aligned with the experiences of dentists in daily clinical practice. The study is guided by four research questions (RQ).

RQ1: Are the reported competences as proposed by academic- and clinical experts also perceived as important by dentists in daily practice?

RQ2: Are the perceptions about required competences by dentists in every day practice influenced by gender and age?

RQ3: What competences are identified as important by dentists in daily practice that are not mentioned in the reported competences proposed by academic- and clinical experts?

RQ4: Considered the development of dentistry over the previous years, how should undergraduate dental education be organised to educate new dentists equipped with the competences to face the challenges in every day practice?

## Methods

In response to the research questions, a questionnaire study was performed to identify the perceptions of Flemish dentists (in the Northern Dutch speaking part of Belgium) about the required competences in daily clinical practice. These perceptions were compared to the competences that were described in the ‘profile and competences for the graduating dentist in Europe’ [11]. Based on academic- and clinical expert opinions this document describes the required competences for graduating European dentists in six domains, including I. professionalism, II. interprofessional, communication and skills, III. knowledge base, information and information literacy, IV. clinical information gathering, V. diagnosis and treatment planning, VI. therapy: establishment and maintaining oral health and VII. prevention and health promotion. In addition, also the dentists’ opinions about the organisation of undergraduate dental education and changes within the profession were addressed.

Based on the research questions, a questionnaire with 28 items was developed in three parts. Part one briefly asked for the participating dentists gender (male or female) and year of birth. Part two described the profile and competences for the graduating European dentist [11] in 21 statements whereby respondents were asked to rate the importance of each statement on a 10-point scale, ranging from totally not important (=1) to indispensable (=10). Additionally, the questionnaire also gauged for any other required competences, not listed in the profile and competences document. The respondents were asked to report any additional required competences to practice dentistry in Flanders. Part three focused on education and included four items. The first item presented 11 domains, based on the Ghent University undergraduate curriculum. These domains included basic sciences, endodontics, evidence based dentistry, gerontology, paediatric dentistry, periodontology, prosthodontics, oral implantology, orthodontics and restorative dentistry. Respondents were asked to rate these items in terms of importance on a 10-point scale, ranging from totally not important (=1) to indispensable (=10). To uncover possible other important domains, respondents also could include a domain in addition to the 11 domains in the questionnaire. The second item asked the respondents to identify the ideal balance between theoretical, preclinical and clinical education in a dental undergraduate curriculum. Respondents had to provide a percentage for all three education types. The third item evaluated the ideal length of an undergraduate dental curriculum as respondents were asked to rate current 5 year programme as too short or long enough or too long. Finally, item 4 assessed remarkable changes in dentistry over the last 15 years. Respondents were asked to mark (yes or no or I don’t know) 11 statements inspired by the experiences in Flanders. They included statements about patients’ assertiveness, shift from individual to group practices, frequency of urgent cases, use of amalgam fillings, shortage of dentists, future shortage of dentists, oral hygiene improvement in patients, need for oral hygienists, need for chairside assistance, increasing administrative load and need for additional training because of increasing innovative growth.

Before distribution, the questionnaire was screened by a panel of 10 dental experts at Ghent University dental school to assess the feasibility to complete the questions, to evaluate the relevance of the items and to identify ambiguity of wording to avoid any misinterpretation. Their comments were used to adjust the questionnaire in terms of wording and the inclusion of statements in multiple items.

The questionnaire (included as Additional file 1) was distributed among dentists in Flanders in the summer of 2015. First, the dentists associated with professional association ‘Verbond der Vlaamse Tandartsen’ (over 3500 members representing 80% of the dentists in Flanders) were approached via a newsletter and were urged to complete the questionnaire online. The newsletter contained a link to the questionnaire that was hosted on the Ghent University online learning environment. A reminder was sent after 3 months. In addition, the questionnaire was parcelled out on paper among the participants of the annual Autumn symposium by the professional association ‘Verbond der Vlaamse Tandartsen’ (*n* = 580). Dentists that had not completed the online questionnaire before were kindly invited to fill in the paper version. The questionnaire was anonymous. The data was analysed using descriptive statistics, Chi-square and non-parametric Mann-Whitney U tests as precaution in the presence of skewed data. All statistical analyses were performed using SPSS 22.0 (IBM SPSS Statistics for Windows, Armonk, NY: IBM Corp.) with a preset significance level of *p* ≤ 0.05. The study protocol was approved by the Ghent University Hospital Ethics Committee under Belgian registration number B670201422622 .

## Results

**Fig. 1:**
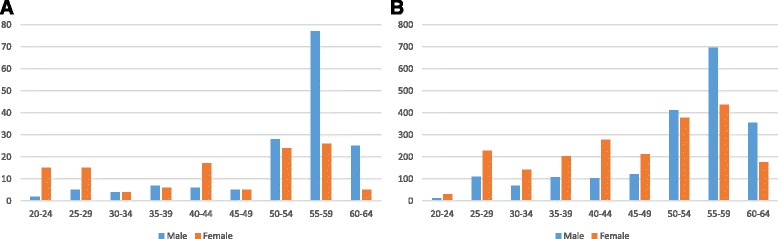
Age distribution related to gender of participating dentists in the study (**a**) as compared to the total Flemish dentist population (**b**). Frequencies are displayed in total amounts for both the dentists in the study as for the total dentist population

A total 312 questionnaires were completed, including 81 online and 231 on paper. In relation to 4836 authorised dentists in Flanders [15] the respondents in the study represented 6.5% of the total dentist population. The respondents had a similar gender- and age distribution compared to the total population. Detailed demographic data on both the respondents as the total dentist population demonstrated a cut-off point at 50 years of age (Fig. 1). Dental professionals under 50 years were characterised by an equilibrium between both genders and with a developing female dominance of new incoming practitioners in the most recent years whereas the majority of dentists of 50 years and above are all male. This cut-off point was used to analyse the impact of age and gender.

All participating dentists perceived the competences mentioned in the ‘profile and competences for the graduating European dentist’ [11] as important to very important as the mean scores ranged between 7.4 and 9.2. Details for each statement are depicted in Table 1.

**Table 1:** The importance of the required competences of the graduating European dentist (11) as perceived by Flemish dentists

Upon graduation a dentist should …	All respondents				<50 years			≥50 years		
	M	SD	SE		M	SD	SE	M	SD	SE
- demonstrate appropriate professional behavior grounded in a wide range of skills required for an adequate management of a dental practice including interaction with patients and colleague health workers, lifelong learning to guarantee optimal care and the creation of a safe work environment for both patients and practitioners. (I)	9.1	1.2	0.07	M F	9.1 9.1	1.0 1.1	0.19 0.14	9.1 9.1	1.1 1.4	0.10 0.19
- demonstrate knowledge and understanding of contemporary laws applicable to the practice of dentistry moral and ethical responsibilities involved in the provision of care to individual patients, populations and communities. (I)	8.5	1.4	0.08	M F	8.3 8.5	1.5 1.2	0.28 0.16	8.5 8.7	1.4 1.6	0.12 0.21
- be competent to communicate effectively, interactively and reflectively with patients, their families, relatives, carers and with other health professionals involved in their care, irrespective of age, social and cultural background. (II)	8.8	1.3	0.08	M F	8.9 8.7	1.2 1.3	0.24 0.16	8.7 8.9	1.2 1.4	0.10 0.19
- be competent to recognise the difference between normal and pathological oral conditions/disorders grounded in the application of knowledge and understanding of basic biological, medical, technical and clinical sciences. (III)	9.2	1.1	0.07	M F	9.3 9.1	1.1 1.2	0.22 0.15	9.1 9.3	1.0 1.3	0.08 0.18
- demonstrate ability to maintain professional knowledge and understanding throughout a professional life. A dentist must demonstrate an appropriate information literacy to acquire and use information in a critical, scientific and effective manner. (III)	8.4	1.4	0.08	M F	8.2 8.0	1.5 1.6	0.28 0.21	8.6 8.5	1.1 1.6	0.10 0.22
- be competent to implement sterilisation, disinfection and antisepsis, and cross-infection control in their practice. (III)	8.9	1.3	0.08	M F	8.9 8.5	1.1 1.6	0.21 0.21	9.0 9.1	1.1 1.3	0.10 0.18
- be competent at obtaining and recording a complete history of the patient’s medical, oral and dental state. (IV)	8.2	1.6	0.09	M F	8.3 8.4	1.7 1.6	0.32 0.21	8.0* 8.6*	1.5 1.6	0.13 0.22
- be competent at performing an appropriate clinical examination; interpreting the findings and organising further investigations when necessary to arrive at an appropriate diagnosis. (IV)	9.0	1.2	0.07	M F	9.2 9.3	1.0 1.0	0.19 0.13	8.9 8.8	1.2 1.4	0.10 0.18
- be competent to identify abnormal and anxiety-related patient behaviour and respond appropriately. (IV)	9.0	1.2	0.07	M F	7.7* 8.5*	1.7 1.3	0.31 0.17	8.1* 8.6*	1.3 1.6	0.11 0.21
- taking radiographs of relevance to dental practice, interpreting the images, including managing and avoiding the hazards of ionising radiation. (IV)	9.0	1.1	0.06	M F	9.1 9.3	1.0 0.9	0.19 0.12	8.9* 9.1*	1.0 1.3	0.09 0.18
- be competent to develop a diagnosis and formulate a treatment plan which meets the needs and demands of patients. (V)	9.1	1.1	0.07	M F	9.2 9.2	1.1 1.0	0.20 0.14	9.0 9.2	1.0 1.4	0.09 0.19
- be competent to identify temporomandibular disorders and associated conditions. (V)	7.9	1.6	0.10	M F	7.4 7.8	1.9 1.6	0.35 0.21	7.8* 8.5*	1.5 1.6	0.13 0.22
- be competent to identify orthodontic treatment need. (V)	8.2	1.5	0.09	M F	7.6 8.3	2.1 1.2	0.39 0.16	8.1* 8.8*	1.5 1.4	0.12 0.19
- be aware of his/her limitations and know when to refer a patient for specialist dental or medical care. (VI)	9.2	1.1	0.06	M F	9.1 9.3	1.2 0.9	0.23 0.11	9.1* 9.3*	0.9 1.4	0.08 0.19
- Treating and managing conditions requiring minor surgical procedures of the hard and soft tissues, and to apply and/or prescribe appropriate pharmaceutical agents to support treatment. (VI)	7.4	1.9	0.12	M F	7.2 6.9	2.1 2.1	0.39 0.27	7.6 7.3	1.8 1.9	0.15 0.26
- know when and how to prescribe antibiotics or other medication. (VI)	9.1	1.1	0.06	M F	9.2 9.2	1.1 0.9	0.20 0.12	9.0 9.2	1.0 1.3	0.09 0.17
- be competent to manage patients with dental urgencies. (VI)	9.0	1.2	0.07	M F	9.2 9.0	1.1 1.2	0.21 0.15	8.9* 9.3*	1.2 1.2	0.11 0.16
- be competent to administer infiltration and block local anaesthesia in the oral cavity and to manage potential complications of local anaesthesia. (VI)	9.0	1.3	0.08	M F	9.2 9.1	1.2 1.1	0.22 0.15	8.9 8.9	1.3 1.6	0.11 0.21
- be competent to carry out basic life support and defibrillation. (VI)	8.3	1.7	0.10	M F	8.0 8.5	1.9 1.5	0.35 0.20	8.0* 8.7*	1.7 1.6	0.15 0.21
- be competent to inform patients about current concepts of prevention, risk assessment and treatment of oral disease which supports the maintenance of systemic and oral health and improves the quality of life for the individual. (VI)	8.5	1.4	0.08	M F	8.2 8.6	1.7 1.4	0.31 0.18	8.5 8.7	1.2 1.5	0.11 0.20
- be competent at promoting and improving the oral health of individuals, families and groups in the community. (VII)	8.2	1.6	0.10	M F	7.9* 8.7*	2.0 1.5	0.37 0.20	8.1 8.1	1.4 1.9	0.12 0.25

The items are scored on a 10-point scale ranging from 1 (= totally not important) to 10 (=indispensable) and displayed in terms of mean (M), standard deviation (SD) and standard error (SE). Results are presented for all respondents, males (M), females (F), < 50 years, and ≥50 years. Each competence is presented in relation to its domain. * *p* ≥ 0.05

In the group of dentists under 50 years, female practitioners rated the importance of identifying and managing anxiety and abnormal behaviour in patients (U = 628.5, z = −2.166, *p* = 0.030); and promoting and improving oral health (U = 627.5, z = −2.192, *p* = 0.028) significantly higher than their male counterparts. In the group of dentists, aged 50 years and above, multiple significant differences were found including, the importance of obtaining and recording a complete history (U = 2759, z = −3.262, *p* ≤ 0.001); identifying and managing anxiety and abnormal behaviour in patients (U = 2888, z = −2.784, *p* = 0.005); obtaining and interpreting radiography in dental practice (U = 3166, z = −2.000, *p* = 0.045); identifying temporomandibular disorders and associated conditions (U = 2765, z = −3.112, *p* = 0.002); identifying orthodontic treatment needs (U = 2664, z = −3.263, *p* ≤ 0.001); being aware of own limitations and knowing when to refer a patient (U = 3087.5, z = −2.442, *p* = 0.015); managing dental urgencies (U = 3113, z = −2.111, *p* = 0.035); and carrying out basic life support and defibrillation (U = 2758, z = −3.038, *p* = 0.002). Each statement was identified as less important by male dentists as compared to female dentists.

In total, 40% of the respondents (*n* = 126) provided additional competences. The vast majority of these additional competences were related to the management of a private dental practice. Furthermore, stress management and empathy were reported. Table 2 provides an overview of identified competences.

**Table 2:** Overview of reported competences by participating dentists in addition to the ADEE competences

Competence	Freq. %
- Management of a private practice	51 (32.7)
- Accountancy and taxation	31 (19.9)
- Stress management	29 (18.6)
- Administration	20 (12.8)
- Informatics	14 (9.0)
- Empathy	11 (7.1)

Results display the frequency (Freq.) and percentage (%)

Part three of the questionnaire focused on the organisation of undergraduate dental education. The respondents identified restorative dentistry, prosthodontics, endodontics, paediatric dentistry and periodontology as most important domains in the curriculum. A detailed overview of all domains is presented in Table 3. In addition to these domains, the respondents also identified business management (*n* = 15) and accounting/taxation (*n* = 10) as important domains to cover in the undergraduate dental curriculum.

**Table 3:** Overview of the relative weight of multiple domains within the undergraduate dental curriculum as suggested by the respondents

Domain	M	SD
Restorative dentistry	9.0	1.2
Prosthodontics	8.8	1.3
Endodontics	8.4	1.5
Paediatric Dentistry	8.0	1.8
Periodontology	7.5	1.7
Gerodontology	7.3	2.0
Oral Implantology	6.8	2.1
Evidence based Dentistry	6.7	2.0
Orthodontics	6.4	2.0
Basic Sciences	6.0	2.0
Management (*n* = 15)^a^	6.8	2.3
Accounting and Taxation (*n* = 10)^a^	6.6	2.3

^a^suggested by respondents including the frequency

Relative weight is expressed on a 10-point scale from 1 = no attention for this domain to 10 = most important domain

Results are displayed by mean (M) and standard deviation (SD)

Concerning the educational strategies, the respondents suggested on average to spend 34% on theoretical education, 26% on preclinical skills training and 40% on clinical practice. The majority of respondents (89%) were in support of an undergraduate curriculum in 5 years. Opposed to this opinion 11% perceived a 5 year curriculum as too short and only one respondent perceived a 5 year curriculum as too long.

All statements were agreed upon by at least 50% of the respondents. Except for the statement about an increase of urgent cases in clinical practice, respondents disagreed. Table 4 presents the agreement percentages of all statements.

**Table 4:** Frequencies (Freq.) and percentages (%) of total respondents (*n* = 312) that agreed with the statements about the evolution of dentistry in the last 15 years

Statement	Freq. %
Significant less amalgam fillings are being placed	281 (90.1%)
A shift is present from individual practice to dentists in group practices	265 (85.0%)
The administrative load has increased	260 (83.3%)
Patients have become more assertive	255 (81.7%)
In the near future there will be a shortage of dentists	228 (73.1%)
The need for chairside assistance has been increased	201 (64.4%)
Innovative grow is increasing causing urgent need for additional training	185 (59.3%)
At present there is a shortage of dentists	166 (53.2%)
Oral hygiene in patients has improved	164 (52.6%)
At present there is a need for dental hygienists	156 (50.0%)
At present more urgent cases are seen in clinical practice	69 (22.1%)

## Discussion

To ensure the provision of high quality oral healthcare, it is essential to educate dentists with the competences to cope with the challenges of every day dental practice. As a result, learning outcomes in dental education should be aligned with the clinical reality of the profession. Findings in the present study illustrate that the proposed competences for the graduating European dentist are also acknowledged by dental practitioners in Flanders (Belgium). Despite this general confirmation of competences, results also identified significant variance in perceived importance of competences between age and gender subgroups. Furthermore, the participating dentists emphasised that professional competences including the management of a private practice, accountancy and taxation, and stress management are important for students to learn. To achieve these competences, dentists in daily clinical practice suggested to develop an undergraduate dental curriculum based on a balanced contribution of clinical education, preclinical skills training and theoretical education.

Multiple profiles for graduating dentists have been proposed, sharing competences in similar domains [14]. Often these profiles have been developed by opinion leaders and governmental organisations. Results in the present study demonstrates that these competences are also confirmed by dental practitioners that experience the clinical reality on a daily basis. Both the similarities between proposed competences around the world and the validation by the clinical field may suggest an underlying global consensus on core competences required in the dental profession.

Nevertheless, results have also illustrated the heterogeneity within the analysed dentist population. The elder generations of dentists were predominantly male. Due to increasing proportion of graduating female dental professionals over the last decades, a feminization of the profession has occurred. The feminization of the dental profession has also been reported in other countries [16, 17]. In the present study, in the 50 years and above dentist group one-third of the competences were rated significantly higher by female dental professionals. In the under 50 years dentist group only two competences were evaluated as significantly more important including the management of anxiety and abnormal behaviour in patients and promoting and improving oral health of individuals, families and groups in the community. Both competences are related to empathy and requires the ability to consider the perspective of others. A trait that is often attributed to female professionals [18].

Faculty has the responsibility to synchronise the undergraduate dental education with the clinical reality. Respondents in the present study identified oral implantology, evidence based dentistry, orthodontics and basic sciences as less important aspects of the undergraduate dental curriculum. Both oral implantology and orthodontics can be identified as advanced specialised care and contain competences that need to be attained at a postgraduate level and hence less justified in undergraduate education. In contrast with the opinion of the respondents, the current perspective on education and dental practice advocates the integration of scientific based practice and evidence based decision making [19]. The integration of basic sciences into the curriculum has its origin in the Flexner report in the previous century. Flexner recommended to introduce a strong foundation of basic sciences in addition to clinical education, enabling students to apply their scientific knowledge in patient care [20, 21]. As a result, education changed from a one-sided clinical approach of internship towards a learning model in which knowledge was taught prior to practical implementation [22]. The respondents in the present study suggested a reduction of basic sciences in favour of applied sciences in various clinical dental domains. This view on education may have been influenced by the current congested undergraduate curriculum in relation to the exponential growing increase in knowledge and understanding. This emphasises the need to continuously evaluate and clearly mark out the undergraduate dental curriculum’s content. Evidence based dentistry is a rather recent phenomenon in dental education. As a result, the dentists in the present study may not fully understand the concept to value its full potential. If this is the case, postgraduate courses including continuing professional development programmes should try to explain dentists the necessity and advantages linked to the evidence based approach.

Furthermore, the results of the present study and the shift from an predominant male profession to an increasing feminization are illustrative of a dynamic society, continuously imposing dentists and curricula to new challenges in order to educate and train dental students to deliver the best healthcare in their future practice and professional life. An important attribute to manage this dynamic context for dental professionals and students is to engage in a process of reflective practice and life-long learning [23–25]. The importance of reflective practice to dentists, however, is not emphasised by the results. This may be the result of a remaining lack of understanding about the implementation of the concept in clinical practice and dental education [25, 26]. Consequently, the concept of reflection may not have been fully permeated among dentists in clinical practices and hence may require extra attention in postgraduate courses.

The present study investigated the competences for the graduating European dentist in terms of generic competence domains. Specific knowledge or skills were not considered. Hence results need to be interpreted in relation to the general perspective of the model. As emphasised by the authors, the European competence profile provides a general blueprint that should be complemented to align with the local context [11]. In addition to the competences in the document, the respondents reported the management of a clinical practice as important competence for undergraduate education, indicating the need to address dentistry as a profession opposed to a single focus on clinical competences.

A questionnaire was used to analyse dental practitioners’ perceptions about required competences in clinical reality. To enable the comparison with the proposed competences for the graduating European dentist, the used competence statements were obtained from the original profile [11]. This approach, however, may also have directed the respondents’ focus solely on these competences, although an additional item gauged for other competences that were not mentioned. Nevertheless, not providing statements and providing open questions as an alternative approach would have troubled the comparison of data. The general positive perceptions about all data may also be influenced by socially desirable answers [27] although this threat was countered by guaranteeing the anonymity of respondents.

A total of 312 questionnaires were completed, representing 6.5% of the population of dentists. In reality this number is probably higher, since the official list with authorised dentists also includes a significant amount of inactive dentists. Furthermore, it needs to be emphasized that the respondents had a similar age and gender distribution as compared to the total population. Hence, this similarity in characteristics may suggest an acceptable study population. In total, the questionnaire contained 28 items. Although the pre-screening suggested an acceptable feasibility to complete the questionnaire, it may have interfered with the response rate.

Future research should focus on unravelling the underlying mechanisms controlling the dynamic and complex relation between societal development, changes in the dental profession and consequences for dental education. Whereas competence profiles are instruments to describe the required competences for dentists, other studies should be directed towards adjusting these competences to a changing context and to provide a translation to convert these profiles into efficient undergraduate dental curricula. A key-factor within this process is to create an environment that facilitates an intensive and continuous exchange between professionals in clinical practice, educators, opinion-leaders and curriculum developers. As a result, educational strategies will be identified in support of learning curves that transform students into dental professionals, able to cope with the challenges of an evolving daily practice.

## Conclusion

Findings in the present study suggest the validation of the proposed required competences for graduating European dentists within the clinical reality of dental professionals in daily practice. Nevertheless, the results have also demonstrated that professional competences including dental practice management, accountancy and taxation or stress-management may be under-exposed. Furthermore, heterogeneity was identified regarding gender and age within the dentist population that may emphasise a continuously evolving dental profession. Hence, to maintain high quality of dental care, a strategy should be developed in which dental curricula are continuously benchmarked against an evolving clinical reality.

## Additional file

Additional file 1: “Questionnaire.pdf” displays the questionnaire that was distributed among dentists in the study. (DOCX 82 kb)

## Abbreviations

RQ: Research Question
