# Supplementary material for: Competence profiles in undergraduate dental education: a comparison between theory and reality
Source: BMC Oral Health. 2017 Jul 11;17:109. doi: 10.1186/s12903-017-0403-4 (PMC5504562; doi:10.1186/s12903-017-0403-4)
Supplement: Additional file 1: — “Questionnaire.pdf” displays the questionnaire that was distributed among dentists in the study. (DOCX 82 kb) [file 12903_2017_403_MOESM1_ESM.docx]

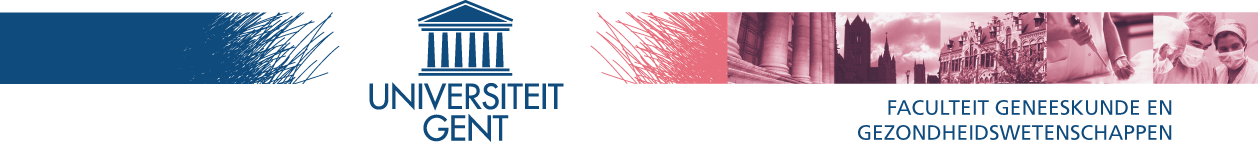


Dear Dentist,

Dentistry is continuously evolving. Scientific and technological progress have caused for many innovations in the past 10 years. Important is that new incoming dentists possess the competences to successfully cope with the challenges in modern dental practice. Hence it is crucial that undergraduate dental education is in line with the evolutions in dentistry.

To investigate this issue, I am doing a masterthesis at Ghent University, under supervision of Sebastiaan Koole, focussed on the perceptions of dental professionals in Flanders about the required competences of graduated dentists.

The research project asks for your cooperation to complete this questionnaire. Working in a dental clinic your opinion is of great importance and you know what is expected from dentists in every day professional practice. We would greatly appreciate 10 minutes of your time to accurately complete this questionnaire. We guarantee your anonymity in the processing, analysing and publication of the data. This research project was approved by the the Ghent University Hospital Ethics Committee

In case of questions or comments please contact: [Shani.VanDenBrulle@UGent.be](mailto:Shani.VanDenBrulle@UGent.be)

We hope for your cooperation and we thank you for your time and effort to complete the questionnaire.

With sincere regards,

Shani Van Den Brulle

Student

Master of Dentistry

Ghent University

Sebastiaan Koole, PhD

Promotor

Ghent University

Department of Dentistry

Periodontology and Oral Implantology

De Pintelaan 185, 1P8

9000 Ghent - Belgium

Email: Sebastiaan.Koole@UGent.be

**Part I: Demographics**

1. Gender:

🞎 Male

🞎 Female

2. Year of birth:

**Part II: Competences required for graduated dentists**

**Which competences are required for graduated dentists to successfully cope with the challenges in dental practice? Rate each statement on a 10-point scale.**

**1 is “totally not important” and 10 is indispensable**

Upon graduation a dentist should …

1. demonstrate appropriate professional behaviour grounded in a wide range of skills required for an adequate management of a dental practice including interaction with patients and colleague health workers, lifelong learning to guarantee optimal care and the creation of a safe work environment for both patients and practitioners.

1 2 3 4 5 6 7 8 9 10

2. demonstrate knowledge and understanding of contemporary laws applicable to the practice of dentistry moral and ethical responsibilities involved in the provision of care to individual patients, populations and communities.

1 2 3 4 5 6 7 8 9 10

3. be competent to communicate effectively, interactively and reflectively with patients, their families, relatives, carers and with other health professionals involved in their care, irrespective of age, social and cultural background.

1 2 3 4 5 6 7 8 9 10

4. be competent to recognise the difference between normal and pathological oral conditions/disorders grounded in the application of knowledge and understanding of basic biological, medical, technical and clinical sciences.

1 2 3 4 5 6 7 8 9 10

5. demonstrate ability to maintain professional knowledge and understanding throughout a professional life. A dentist must demonstrate an appropriate information literacy to acquire and use information in a critical, scientific and effective manner.

1. 2 3 4 5 6 7 8 9 10

6. be competent at obtaining and recording a complete history of the patient’s medical, oral and dental state.

1 2 3 4 5 6 7 8 9 10

7. be competent to develop a diagnosis and formulate a treatment plan which meets the needs and demands of patients.

1 2 3 4 5 6 7 8 9 10

8. be competent at performing an appropriate clinical examination; interpreting the findings and organising further investigations when necessary to arrive at an appropriate diagnosis.

1 2 3 4 5 6 7 8 9 10

9. be aware of his/her limitations and know when to refer a patient for specialist dental or medical care.

1 2 3 4 5 6 7 8 9 10

10. be competent to inform patients about current concepts of prevention, risk assessment and treatment of oral disease which supports the maintenance of systemic and oral health and improves the quality of life for the individual.

1 2 3 4 5 6 7 8 9 10

11. treating and managing conditions requiring minor surgical procedures of the hard and soft tissues, and to apply and/or prescribe appropriate pharmaceutical agents to support treatment.

1. 2 3 4 5 6 7 8 9 10

12. be competent to identify abnormal and anxiety-related patient behaviour and respond appropriately.

1 2 3 4 5 6 7 8 9 10

13. be competent at promoting and improving the oral health of individuals, families and groups in the community.

1 2 3 4 5 6 7 8 9 10

14. taking radiographs of relevance to dental practice, interpreting the images, including managing and avoiding the hazards of ionising radiation.

1 2 3 4 5 6 7 8 9 10

15. know when and how to prescribe antibiotics or other medication.

1. 2 3 4 5 6 7 8 9 10

16. be competent to manage patients with dental urgencies.

1 2 3 4 5 6 7 8 9 10

17. be competent to administer infiltration and block local anaesthesia in the oral cavity and to manage potential complications of local anaesthesia.

1. 2 3 4 5 6 7 8 9 10

18. be competent to identify temporomandibular disorders and associated conditions.

1. 2 3 4 5 6 7 8 9 10

19. be competent to identify orthodontic treatment need.

1 2 3 4 5 6 7 8 9 10

20. be competent to carry out basic life support and defibrillation.

1. 2 3 4 5 6 7 8 9 10

21. be competent to implement sterilisation, disinfection and antisepsis, and cross-infection control in their practice.

1 2 3 4 5 6 7 8 9 10

Report any additional required competences that are not mentioned in the statements above:

________________________________________________________________________________________________________________________________________________________________________________________________________________________________________________________________________________________________________________________________________

**Part III: Education**

**Please rate on a 10-point scale how many time should be spent in undergraduate dental education on the domains below?**

**1= no time should be spent on this domain**

**10= the complete curriculum should be spent on this domain**

Basic sciences:

1 2 3 4 5 6 7 8 9 10

Evidence-based dentistry:

1 2 3 4 5 6 7 8 9 10

Restorative dentistry:

1 2 3 4 5 6 7 8 9 10

Prosthodontics:

1 2 3 4 5 6 7 8 9 10

Endodontics:

1 2 3 4 5 6 7 8 9 10

Paediatric dentistry:

1 2 3 4 5 6 7 8 9 10

Orthodontics:

1 2 3 4 5 6 7 8 9 10

Periodontology:

1 2 3 4 5 6 7 8 9 10

Oral implantology:

1 2 3 4 5 6 7 8 9 10

Gerontology:

1 2 3 4 5 6 7 8 9 10

Other:__________________________

1 2 3 4 5 6 7 8 9 10

**How many time should be spent in undergraduate dental education on the educational strategies below? Please provide a percentage.**

|  | percentage |
| --- | --- |
| Theoretical education | % |
| Preclinical education | % |
| Clinical education | % |
| Total | 100 % |

**The current undergraduate dental curriculum in Flanders is 5 years. What is your opinion about this?**

🞎 Too short

🞎 Long enough

🞎 Too long

**What has changed in the past 15 years in dental practice?**

| **Do you think that:** | **Yes** | **No** | **I don’t know** |
| --- | --- | --- | --- |
| 1. patients have become more assertive | 🞎 | 🞎 | 🞎 |
| 1. a shift is present from individual practice to dentists in group practices | 🞎 | 🞎 | 🞎 |
| 1. at present more urgent cases are seen in clinical practice | 🞎 | 🞎 | 🞎 |
| 1. significant less amalgam fillings are being placed | 🞎 | 🞎 | 🞎 |
| 1. at present there is a shortage of dentists | 🞎 | 🞎 | 🞎 |
| 1. in the near future there will be a shortage of dentists | 🞎 | 🞎 | 🞎 |
| 1. oral hygiene in patients has improved | 🞎 | 🞎 | 🞎 |
| 1. at present there is a need for dental hygienists | 🞎 | 🞎 | 🞎 |
| 1. the need for chairside assistance has been increased | 🞎 | 🞎 | 🞎 |
| 1. the administrative load has increased | 🞎 | 🞎 | 🞎 |
| 1. innovative grow is increasing and is causing an urgent need for additional training | 🞎 | 🞎 | 🞎 |

Thank you for your cooperation!
